# No Difference in Lactoferrin Levels between Metabolically Healthy and Unhealthy Obese Women

**DOI:** 10.3390/nu11091976

**Published:** 2019-08-22

**Authors:** Małgorzata Jamka, Patrycja Krzyżanowska-Jankowska, Edyta Mądry, Aleksandra Lisowska, Paweł Bogdański, Jarosław Walkowiak

**Affiliations:** 1 Department of Pediatric Gastroenterology and Metabolic Diseases, Poznan University of Medical Sciences, Szpitalna Str. 27/33, 60-572 Poznań, Poland; 2 Department of Physiology, Poznan University of Medical Sciences, Święcickiego Str. 6, 60-781 Poznań, Poland; 3 Department of Treatment of Obesity, Metabolic Disorders and Clinical Dietetics, Poznan University of Medical Sciences, Szamarzewskiego Str. 84, 60-569 Poznań, Poland

**Keywords:** lactoferrin, metabolically healthy obesity, metabolically unhealthy obesity, metabolic syndrome, obesity

## Abstract

Background: The aim of the study was to compare serum lactoferrin concentrations in metabolically healthy obese (MHO) and metabolically unhealthy obese (MUHO) women. Methods: Three hundred (101 MHO and 199 MUHO) women were recruited to the study. Basic anthropometric parameters and blood pressure were measured. Body mass index (BMI) was calculated. Fat mass and visceral adipose tissue mass were assessed using dual X-ray absorptiometry scan. Fasting glucose, insulin, lipid profile, high sensitivity C-reactive protein (hs-CRP) and lactoferrin levels were determined. Results: Lactoferrin levels did not differ between MHO and MUHO subjects (median (interquartile range): 1639 (1055–2396) vs. 1622 (1009–23345) ng/mL). However, in the total population insulin (r = 0.131, *p* = 0.0234) and hs-CRP (r = 0.165, *p* = 0.0045) levels were correlated with lactoferrin concentrations. In addition, a weak positive association between serum lactoferrin concentrations and anthropometric parameters was also detected, and predominantly referred to MHO group (body weight: r = 0.231, *p* = 0.0201; BMI: r = 0.286, *p* = 0.0037; waist circumference: r = 0.258, *p* = 0.0092). In addition, serum lactoferrin concentrations were negatively correlated with fasting glucose (r = −0.250, *p* = 0.0115) and HDL-C levels (r = −0.203, *p* = 0.0411) in MHO subjects. Conclusions: Lactoferrin levels did not differ between MHO and MUHO women. However, some mild correlations between lactoferrin concentrations and anthropometric and metabolic parameters were observed mostly in MHO subjects.

## 1. Introduction

Obesity is an important worldwide public health problem, which increases the risk of development of metabolic abnormalities [1]. It is estimated that around 75% of obese subjects suffer from metabolic syndrome. However, obesity is not always associated with metabolic abnormalities [2]. It is assumed that prevalence of metabolically healthy obesity ranges between 3.3 and 32.1% in men and between 11.4 and 43.3% in women according to the diagnostic criteria applied for definition. Unfortunately, to date, factors related to healthy and unhealthy obesity phenotypes remain undetermined [3].

Lactoferrin is an iron-binding glycoprotein which is present in high concentrations in human and bovine milk, as well as in lower amounts in exocrine secretions (e.g., saliva, tears, semen, vaginal fluids, gastrointestinal fluids) and cells (e.g., neutrophils, leukocytes, enterocytes and adipocytes). Lactoferrin receptors (low-density lipoprotein receptor-related protein 1 and 2, intelectin 1 and nucleolin) are localised on a variety of cells such as monocytes, lymphocytes, adipocytes, hepatocytes and endothelial cells [4]. So far, multiple activities of lactoferrin have been shown in both preclinical [5,6,7] and clinical studies [8,9]. It was documented that lactoferrin possesses anti-inflammatory, antibacterial, antiviral, immunomodulatory, antioxidant, and anticancer activities [4]. In addition, lactoferrin was shown to lower inflammation, oxidative stress and apoptosis, which are key mechanisms involved in the progression of various cardiometabolic abnormalities [10]. Lactoferrin also down-regulated proinflammatory cytokine production in cell lines acting via nuclear factor-α, leading to decreased release of tumor necrosis factor α and interleukin-6 in mice [11]. 

Moreno–Navarrete et al. [12] also proposed that endogenous lactoferrin biosynthesis is essential to achieving adequate adipogenesis. In this context, it is supposed that circulating lactoferrin concentrations might be significantly associated with anthropometric parameters. Indeed, a negative correlation between circulating lactoferrin concentrations and body mass index (BMI) was noted [8]. Lactoferrin has also been described to display beneficial effects on plasma lipid levels. Moreno–Navarrete et al. [8] reported inverse correlations between lactoferrin levels and serum TG concentrations, speculating that the preservation of lactoferrin production leads to decreased free lipopolysaccharide concentrations, thereby maintaining an adequate lipid profile [8,9]. It was also suggested that lactoferrin might promote cholesterol excretion via interactions with bile acids [6]. 

A negative correlation between circulating lactoferrin concentrations and fasting glucose concentrations and a positive correlation between circulating lactoferrin levels and insulin sensitivity was documented [13]. It has been also shown that lactoferrin levels were significantly lower in patient with impaired glucose tolerance compared to normal glucose tolerance patients [8]. It is possible that lactoferrin has a direct impact on insulin resistance in peripheral organs. Indeed, lactoferrin was shown to improve the insulin-signaling response in mature adipocytes through the increase of Protein Kinase B (AKT) serine 473 phosphorylation and through an improvement in the expression of glucose transport 4 and insulin receptor [13]. Finally, lactoferrin-derived peptide may have a potent antihypertensive function notably by blocking the angiotensin AT1 receptor and by inhibiting the renin-angiotensin and the endothelin system [14]. 

Recent studies have demonstrated that lactoferrin might modulate lipid metabolism, glucose and insulin homeostasis and lactoferrin levels are correlated with anthropometric parameters. However, this association was observed mostly in male population [8,9], while it cannot be ruled out that lactoferrin levels are differentially affected metabolic parameters in men and women. Indeed, Kim et al. [15] conducted a study in Latino youth subjects and showed higher obesity risk accompanies higher lactoferrin concentrations for men. However, there were no significant associations between lactoferrin levels and obesity-related phenotypes in women. Given the fact that lactoferrin levels may vary across sex, it is necessary to confirm previous findings in women populations [16].

Therefore, the aim of the study was to compare serum lactoferrin concentrations in metabolically healthy obese (MHO) and metabolically unhealthy obese (MUHO) women, also examining the potential correlation between lactoferrin levels and individual components of the metabolic syndrome.

## 2. Materials and Methods

### 2.1. Study Population

Adult women with abdominal obesity (BMI ≥ 30 kg/m^2^ and waist circumference ≥ 88 cm) were recruited to the study among patients of medical clinics and medical centers in the Wielkopolska region, in consultation with their doctors and directors of the clinics. Before the start of the study, the potential subjects were screened by a dietician during an inclusion visit to comply with protocol requirements. Participants were included in MHO and MUHO groups based on the National Cholesterol Education Program (NCEP) Adult Treatment Panel III (ATP III) criteria. MHO subjects had less than three of the following disorders, while MUHO subjects possessed at least three of the following abnormalities: waist circumference: ≥88 cm; systolic blood pressure ≥ 130 mmHg and/or diastolic blood pressure ≥ 85 mmHg or current use of antihypertensive drugs; triglyceride levels ≥ 150 mg/dL (1.7 mmol/L); fasting glucose levels ≥ 100 mg/dL (5.6 mmol/L); HDL-C levels < 50 mg/dL (1.29 mmol/L) [17]. Exclusion criteria included history of use of any dietary supplements within three months before the study, cancer diagnosis in the last five years, acute and chronic kidney or liver diseases, systemic inflammatory diseases and any other serious diseases, general poor health status, smoking, pregnant and breastfeeding women.

Study participants received information about the study and were informed that participation was voluntary and that they could withdraw at any time without providing reasons. Written informed consent was obtained from all subjects. The present study was conducted according to the guidelines of the Declaration of Helsinki. The protocol was approved by the Poznan University of Medical Sciences Bioethical Committee for the ethical treatment of subjects participating in biomedical research (refs. 984/17).

### 2.2. Sample Size Calculation

G*Power 3.1.9.2 software (University of Kiel, Kiel, Germany) was used to calculate the minimum sample size based on differences in lactoferrin levels in subjects with and without insulin resistance, as previously reported by Mayeur et al. [18]. The sample size required to obtain a power of 95% (α = 0.05, β = 0.05) was 96 subjects (48 subjects per group); assuming that 10% of patients may withdraw from the study, a minimum of 54 subjects should be recruited for each group.

### 2.3. Anthropometry Parameters

Anthropometry was assessed using body height, body weight and waist circumference. During anthropometric measurements, all participants were dressed in light clothing and were barefoot. Waist circumference was measured on bare skin at the level of the iliac crest with the subjects at minimal respiration. In this study, abdominal obesity was according to the NCEP criteria with a waist circumference exceeding 88 cm in women [17]. BMI was calculated as body weight in kilograms divided by body height in meters squared. The BMI classification according to the World Health Organization criteria was used [19].

### 2.4. Body Composition

Body composition was assessed using a dual-energy X-ray absorptiometry (DXA) with the application of the Hologic Discovery DXA system (Bedford, MA, USA). Based on the examination, fat mass (FM), fat-free mass (FFM) and visceral adipose tissue (VAT) mass were measured. The American Society of Bariatric Physicians’ criteria of body fat ≥ 30% for women was used for the diagnosis of obesity [20].

### 2.5. Blood Pressure

Blood pressure was measured before blood sample collection, according to guidelines of the European Society of Hypertension based on the oscillometric method using a portable sphygmomanometer. Blood pressure was assessed on the arm at heart level and was expressed as systolic (SBP) and diastolic pressures (DPB) [21]. The average of three measurements was used for statistical analysis.

### 2.6. Blood Collection

Blood samples were drawn from the antecubital vein via standard venepuncture by registered staff nurses. Blood samples were taken from the participants after 12 h of fasting and stored at −80 °C until the day of analysis.

### 2.7. Biochemical Measurements

Biochemical analyses included the following parameters: lactoferrin, fasting glucose, insulin, TC, LDL-C, HDL-C, TG and high-sensitivity C-reactive protein (hs-CRP) levels. All biochemical parameters were measured with standard clinical chemical assays. Serum lactoferrin concentrations were assessed with well-established commercial enzyme-linked immunosorbent assay (ELISA) kit (BIOXYTECH Lactof EIA reagent set, Oxis Research, Oxis International, Beverly Hills, CA, USA). Insulin resistance was evaluated by calculating the homeostatic model assessment of insulin resistance (HOMA-IR) index based on the following formula: HOMA-IR = [glucose (mg/dL) × insulin (μU/mL)/405] using fasting glucose values [22]. According to ATP III, the cut-off values of HOMA-IR for the diagnosis of insulin resistance was ≥1.8 [23].

### 2.8. Statistical Analysis

STATISTICA 13.0 software (StatSoft, Inc., Cracow, Poland) was used for statistical analyses. A two-sided *p* < 0.05 was regarded as statistically significant. The variables were tested for normality based on the Shapiro–Wilk test. The characteristics of the cohort were expressed as medians and the interquartile ranges (IQR) based on non-parametric data distribution. Comparisons between two unpaired groups were conducted using the Mann–Whitney U test, with Spearman coefficients correlations calculated to evaluate the relationships between analyzed parameters separately for MHO and MUHO groups. Univariate linear regression analysis was subsequently performed to assess the relationship between serum lactoferrin concentrations and age, BMI, waist circumference, FM, VAT mass, SBP, DBP, glucose, insulin, TC, LDL-C, HDL-C, TG, hs-CRP levels and HOMA-IR in the total population and separately in MHO and MUHO groups. Variables from univariate analysis with *p* < 0.1 were subsequently entered into the multivariate linear regression analysis.

## 3. Results

### 3.1. Study Cohort

Table 1 summarizes the clinical characteristics of the study population. In total, 300 non-smoking women (101 MHO and 199 MUHO) with abdominal obesity were recruited to the study with a median age of 57 (IQR: 52–62) years.

Table 2 compares MHO and MUHO group. As expected, MHO subjects displayed significantly (*p* < 0.05) lower waist circumference, SBP, glucose, insulin, TG levels and HOMA-IR index, whereas serum HDL-C concentrations were significantly higher in comparison to the MUHO group. However, there were no significant differences in serum lactoferrin concentrations between MHO and MUHO subjects: 1639 (IQR: 1055–2396) versus 1622 (IQR: 1009–23,345) ng/mL (*p* > 0.05) also after adjustment for: (1) BMI and hs-CRP; (2) waist circumference and hs-CRP; (3) FM and hs-CRP; (4) VAT mass and hs-CRP.

### 3.2. Association between Lactoferrin Levels and Metabolic Parameters

Correlations between lactoferrin levels and selected variables are presented in Table 3. There was a weak positive association between serum lactoferrin concentrations and anthropometric parameters: body weight, BMI, waist circumference, FM in the total population (Supplementary Figures S1–S4). In addition, a positive correlation was observed between lactoferrin levels and insulin concentrations (Supplementary Figure S5) as well as lactoferrin concentrations and hs-CRP levels (Supplementary Figure S6). In MHO subjects, there was a significant association between anthropometric parameters and lactoferrin levels (Supplementary Figures S7–S10). Moreover, in this group lactoferrin levels were also negatively correlated with fasting glucose (Supplementary Figure S11) and HDL-C levels (Supplementary Figure S12). In MUHO subjects, there was only a positive association between lactoferrin concentrations and waist circumference (Supplementary Figure S13), as well as between lactoferrin levels and hs-CRP concentrations (Supplementary Figure S14).

Table 4 shows that in total population BMI, waist circumference and VAT mass were significantly associated with lactoferrin levels. In MHO group we observed the relationship between BMI, VAT mass, HDL-C concentrations and lactoferrin levels, while in MUHO group BMI and waist circumference were associated with serum lactoferrin concentrations. Multivariate linear regression was performed in the total population and showed that BMI, waist circumference, VAT mass and hs-CRP contributed independently to lactoferrin levels (Table 5).

## 4. Discussion

Only few studies have so far attempted to define the associations between lactoferrin concentrations and metabolic parameters. In these studies, lactoferrin levels were compared in lean and severely obese participants, diabetic and non-diabetic patients, insulin resistant and insulin sensitive subjects [18], as well as in persons with altered and normal glucose tolerance [9]. However, this is the first study that compared serum lactoferrin concentrations in MHO and MUHO subjects. Here, we did not observe any significant difference in lactoferrin levels between groups. Nevertheless, there was a weak association between anthropometric parameters and serum lactoferrin concentrations that in fact predominantly was related to MHO subjects. Lactoferrin levels were also negatively correlated with fasting glucose and HDL-C levels in MHO subjects and positively correlated with insulin concentrations in the total population and with hs-CRP levels especially in MUHO subjects.

The association between lactoferrin levels and anthropometric parameters was previously suggested by Moreno–Navarette et al. [9,24], who observed lower lactoferrin levels in moderately obese subjects compared to overweight participants [24] and noted that plasma lactoferrin concentrations are negatively correlated with BMI and waist to hip ratio in subjects with altered glucose tolerance [9]. Mayeur et al. [18] also revealed negative correlations between plasma lactoferrin concentrations and adiposity indices (BMI and FM) in lean to moderately obese women admitted for gynaecological surgery. In fact, subjects with elevated values of BMI, FM or abdominal adiposity, had reduced plasma lactoferrin concentrations compared to leaner subjects. Subcutaneous and visceral adipose tissue showed similar correlation coefficients, suggesting that total adiposity, rather than the specific fat distribution pattern, was the main correlate of lactoferrin levels, whereas Kim et al. [16] reported a positive association between BMI, FM, waist and hip circumferences and lactoferrin levels in youth, non-diabetic, Latino males. To explain the obtained results, authors proposed that in adults, hyperglycaemia or inflammatory responses could interrupt degranulation of neutrophils, thus reduce lactoferrin secretion efficiency, while in children inflammation-related metabolic status might be less impacted compared to adults. 

In this study, serum lactoferrin concentrations were positively correlated with anthropometric parameters (body weight, BMI, waist circumference and VAT mass) in obese subjects, which is discordant with previous reports. Indeed, studies in adult populations showed a negative correlation between lactoferrin levels and anthropometric parameters [9,18]. It is probable that the inconsistencies in obtained results might be related to the increasing popularity of lactoferrin supplements in recent years. It could also be attributed to the concomitant treatments of these subjects (with statins, fibrates, insulin, and oral antidiabetic drugs) [8]. It is well-known that neutrophils are increased in obesity, but their capacity to release antimicrobial proteins are attenuated, mainly in association with insulin resistance, which could explain the positive correlation between serum lactoferrin and obesity measures.

Moreover, lactoferrin levels were positively correlated with insulin concentrations, in line with a previous reported by Mayeur et al. [18], who found that plasma lactoferrin levels were positively correlated with fasting insulin concentrations and HOMA-IR index in a group of lean to moderately obese women and in the cohort of non-diabetic and severely obese subjects. The precise mechanisms behind the increase in plasma lactoferrin concentrations in subjects with high insulin levels have not been defined. Studies have suggested a regulatory role of insulin in circulating lactoferrin induction, however, the impact of additional factors cannot be excluded, such as inflammation and oxidative stress, which can deteriorate insulin sensitivity, thereby increasing insulin levels. Nevertheless, lactoferrin may exert a significant influence on insulin signaling and related functions, including improvement of Akt serine 473 phosphorylation, an increase in glucose transporter type 4 and insulin receptor expression in mature adipocytes, enhancement of the glucose disappearance rate and reduction in inflammation and oxidative stress [13,18].

The present study also showed a negative correlation between fasting glucose concentrations and lactoferrin levels in MHO subjects. Previously, similar results were demonstrated in several [8,9,25], albeit not all [15,26], studies. In addition, this correlation was described in patients with altered glucose metabolism but not in subjects with non-pathological glucose levels [8,9]. It is possible that lactoferrin might modulate glucose absorption from the small intestine, thus impact on glucose levels. Indeed, Maekawa et al. [27] reported that lactoferrin could suppress hyperglycaemia, accompanied by elevated plasma levels of insulin via transiently accelerating glucagon-like peptide-1 secretion, thereby enhancing glucose absorption. Nevertheless, other studies are needed to explain the potential association between lactoferrin levels and glucose homeostasis.

In MHO subjects, serum lactoferrin concentrations were also negatively correlated with HDL-C levels. However, these findings are in contrast to previous results. Mayeur et al. [18] reported that HDL-C levels were not associated with plasma lactoferrin concentrations in lean to moderately obese women. In addition, Fernández–Real et al. [25] observed that baseline circulating lactoferrin concentrations did not correlate with HDL-C levels. Similar results were observed in children [26]. In contrast, Moreno–Navarrete et al. [9] showed that plasma lactoferrin concentrations were directly associated with HDL-C concentrations. These associations were strengthened in subjects with altered glucose tolerance. 

Several mechanisms may explain the association between lactoferrin levels and HDL-C concentration reported herein. In fact, bovine lactoferrin was found to reduce the accumulation of cholesteryl esters in macrophages incubated with acetylated LDL by more than 80% compared with the control. Treatment with bovine lactoferrin reportedly also leads to decreased intestinal absorption of TG via lymphatic pathways [7,28].

Although recent studies have demonstrated that lactoferrin might modulate lipid metabolism, glucose and insulin homeostasis [8,9], no differences in serum lactoferrin concentrations were observed between MHO and MUHO subjects in this study. In addition, serum lactoferrin concentrations were not associated with TC, LDL-C and TG levels or blood pressure. Several factors might impact on the obtained results. For instance, lactoferrin levels are influenced by lifestyle factors, such as diet and experimental data have shown that diet represents a significant source of exogenous lactoferrin that can contribute to its availability in the gastrointestinal tract and blood circulation [7,29]. However, eating habits were not systematically considered here, thus limiting our findings. In addition, according to recent studies, variants in several genes involved in lactoferrin metabolism might be related to the risk of developing metabolic abnormalities. Indeed, lactoferrin gene polymorphisms (*LTF* rs1126477 and rs1126478) were reported to be associated with HDL-C and TG levels in subjects with altered glucose tolerance [8]. In addition, it was previously reported that a polymorphism in the lactoferrin receptor gene (low-density lipoprotein receptor-related protein 1 (*LRP1*) rs4759277) was associated with fasting insulin levels and HOMA-IR [30]. 

Atherosclerosis and cardiac disease are also associated with increased cardiovascular inflammation, specifically as measured by hs-CRP levels [31]. Previously, Fernández-Real et al. [25] observed in severely obese patients an inverse association between lactoferrin levels and hs-CRP concentrations, both at baseline and after a fat overload. Conversely, Marcil et al. [26] did not show an association between lactoferrin levels and hs-CRP concentrations. Here, we noted that serum lactoferrin concentrations were positively associated with hs-CRP levels, mostly in MUHO group. It is widely known that lactoferrin possess immunomodulatory function. Therefore, higher levels of lactoferrin might be synthesized in response to the inflammatory process. Nevertheless further studies are needed to decline this association [32].

Although Vengen et al. [33] showed a strong correlation between parallel measurements of lactoferrin in serum and plasma, Adeyemi and Hodgson [34] reported that the release of lactoferrin from neutrophils during blood clotting could result in the overestimation of serum lactoferrin concentrations in comparison to plasma samples. Indeed, serum lactoferrin concentrations reported here were significantly higher than plasma lactoferrin concentrations in other studies [8,14,18]. Moreover, neutrophils levels and inflammatory markers were not assessed, that might be significantly associated with lactoferrin levels. 

Nonetheless, the strengths of this study include high homogeneity and a well-characterized study population, with clear inclusion and exclusion criteria. In addition, to the best of our knowledge, this is the first study that compared serum lactoferrin concentrations in MHO and MUHO subjects.

## 5. Conclusions

This study demonstrated serum lactoferrin concentrations did not differ between MHO and MUHO women. However, some mild correlations between lactoferrin levels and anthropometric and metabolic parameters were observed mostly in MHO subjects.

## Figures and Tables

**Table 1:** Clinical characteristics of study population (*n* = 300).

	Median (IQR)
Age (years)	57 (52–62)
Height (cm)	161 (157–165)
Weight (kg)	90.4 (82.4–101.6)
BMI (kg/m^2^)	35.16 (31.83–38.14)
Waist circumference (cm)	108 (103–115)
FM (g)	38151 (32,091–44,026)
FM (%)	43.1 (38.9–47.0)
VAT mass (g)	1065 (883–1274)
SBP (mmHg)	138 (129–149)
DPB (mmHg)	85 (79–90)
Glucose (mg/dL)	97 (90–106)
Insulin (µU/mL)	13.05 (8.80–17.70)
HOMA-IR	3.00 (2.12–4.35)
TC (mg/dL)	219 (186–246)
LDL-C (mg/dL)	134 (107–160)
HDL-C (mg/dL)	54 (45–62)
TG (mg/dL)	135 (101–178)
hs-CRP (mg/L)	3.10 (1.50–5.90)
Lactoferrin (ng/mL)	1628 (1019–2374)

**Table 2:** Comparison of metabolically healthy obese (MHO) and metabolically unhealthy obese (MUHO) groups.

	MHO (*n* = 101)	MUHO (*n* = 199)	*p*
Age (years)	57 (52–62)	58 (52–63)	0.4730
Height (cm)	161 (157–165)	161 (157–166)	0.5124
Weight (kg)	90.7 (82.0–100.7)	90.4 (83.5–101.9)	0.2934
BMI (kg/m^2^)	34.30 (31.90–37.40)	35.36 (31.79–38.85)	0.2126
Waist circumference (cm)	107 (99–113)	109 (104–116)	0.0112
FM (g)	37487 (31,697–43,868)	38690 (32,586–44,620)	0.2638
FM (%)	42.6 (38.9–47.2)	43.5 (38.9–47.0)	0.3945
VAT mass (g)	1035 (877–1230)	1072 (887–1314)	0.1998
SBP (mmHg)	131 (125–140)	140 (131–152)	<0.0001
DPB (mmHg)	83 (78–88)	85 (79–93)	0.0539
Glucose (mg/dL)	93 (89–96)	101 (92–109)	<0.0001
Insulin (µU/mL)	10.10 (7.10–14.60)	14.20 (10.60–19.00)	<0.0001
HOMA-IR	2.35 (1.64–3.31)	3.38 (2.49–5.10)	<0.0001
TC (mg/dL)	220 (184–244)	219 (190–247)	0.5621
LDL-C (mg/dL)	135 (99–158)	133 (109–161)	0.5156
HDL-C (mg/dL)	60 (56–71)	48 (43–57)	<0.0001
TG (mg/dL)	106 (86–122)	155 (120–199)	<0.0001
hs-CRP (mg/L)	2.85 (1.45–5.15)	3.35 (1.60–6.35)	0.2481
Lactoferrin (ng/mL)	1639 (1055–2396)	1622 (1009–2335)	0.5303
Lactoferrin (ng/mL) adjusted for BMI and hs-CRP	1790 (1667–1915)	1809 (1653–1981)	0.3440
Lactoferrin (ng/mL) adjusted for waist circumference and hs-CRP	1780 (1646–1954)	1823 (1691–1981)	0.0900
Lactoferrin (ng/mL) adjusted for FM and hs-CRP	1882 (1777–2006)	1898 (1794–2009)	0.4406
Lactoferrin (ng/mL) adjusted for VAT mass and hs-CRP	1815 (1725–1956)	1867 (1739–2033)	0.0944

**Table 3:** Correlations between lactoferrin levels and selected variables.

	Total Population (*n* = 300)		MHO (*n* = 101)		MUHO (*n* = 199)	
	r	*p*	r	*p*	r	*p*
Age (years)	−0.058	0.3132	−0.013	0.8981	−0.081	0.2545
Weight (kg)	0.161	0.0052	0.231	0.0201	0.119	0.0931
BMI (kg/m^2^)	0.187	0.0011	0.286	0.0037	0.137	0.0529
Waist circumference (cm)	0.186	0.0012	0.258	0.0092	0.166	0.0191
FM (g)	0.121	0.0488	0.177	0.0911	0.095	0.2083
FM (%)	0.087	0.1565	0.133	0.2052	0.057	0.4523
VAT mass (g)	0.115	0.0550	0.246	0.0162	0.054	0.4668
SBP (mmHg)	−0.018	0.7563	−0.004	0.9654	−0.010	0.8840
DPB (mmHg)	−0.072	0.2142	−0.005	0.9574	−0.091	0.2015
Glucose (mg/dL)	−0.108	0.0620	−0.250	0.0115	−0.079	0.2640
Insulin (µU/mL)	0.131	0.0234	0.190	0.0573	0.132	0.0630
HOMA-IR	0.101	0.0806	0.168	0.0937	0.097	0.1724
TC (mg/dL)	−0.039	0.5042	−0.027	0.7889	−0.047	0.5068
LDL-C (mg/dL)	−0.028	0.6276	0.046	0.6443	−0.065	0.3614
HDL-C (mg/dL)	−0.046	0.4242	−0.203	0.0411	−0.027	0.7066
TG (mg/dL)	0.037	0.5244	0.047	0.6380	0.032	0.6477
hs-CRP (mg/L)	0.165	0.0045	0.184	0.0672	0.159	0.0262

**Table 4:** Univariate regression analysis assessing the relationship between serum lactoferrin concentrations (ng/mL) and selected variables.

	Total Population (*n* = 300)				MHO (*n* = 101)				MUHO (*n* = 199)			
	β	SE	t	*p*	β	SE	t	*p*	β	SE	t	*p*
Age (years)	−0.065	0.058	−1.133	0.2582	−0.019	0.100	−0.189	0.8503	−0.083	0.071	−1.176	0.2410
BMI (kg/m^2^)	0.197	0.057	3.465	0.0006	0.256	0.097	2.631	0.0098	0.174	0.070	2.482	0.0139
Waist circumference (cm)	0.187	0.057	3.287	0.0011	0.192	0.099	1.947	0.0544	0.193	0.070	2.755	0.0064
Fat mass (%)	0.103	0.061	1.680	0.0940	0.115	0.105	1.097	0.2757	0.098	0.076	1.291	0.1982
VAT mass (g)	0.138	0.059	2.318	0.0212	0.267	0.100	2.671	0.0089	0.088	0.074	1.194	0.2340
SBP (mmHg)	−0.007	0.058	−0.122	0.9031	−0.053	0.100	−0.528	0.5989	0.021	0.071	0.290	0.7722
DPB (mmHg)	−0.051	0.058	−0.882	0.3783	−0.027	0.100	−0.269	0.7887	−0.059	0.071	−0.834	0.4051
Glucose (mg/dL)	−0.048	0.058	−0.824	0.4105	−0.124	0.100	−1.249	0.2145	−0.022	0.071	−0.308	0.7585
Insulin (µU/mL)	0.081	0.058	1.399	0.1628	0.142	0.099	1.423	0.1577	0.072	0.071	1.017	0.3103
HOMA-IR	0.061	0.058	1.056	0.2917	0.116	0.100	1.165	0.2466	0.058	0.071	0.817	0.4151
TC (mg/dL)	−0.052	0.058	−0.902	0.3675	−0.046	0.100	−0.456	0.6491	−0.053	0.071	−0.748	0.4550
LDL-C (mg/dL)	−0.057	0.058	−0.989	0.3234	0.021	0.100	0.205	0.8380	−0.090	0.071	−1.269	0.2060
HDL-C (mg/dL)	−0.089	0.058	−1.550	0.1221	−0.222	0.098	−2.264	0.0257	−0.065	0.071	−0.918	0.3595
TG (mg/dL)	0.021	0.058	0.370	0.7119	0.001	0.100	0.011	0.9914	0.040	0.071	0.561	0.5752
hs-CRP (mg/L)	0.110	0.058	1.898	0.0586	0.088	0.101	0.880	0.3811	0.123	0.071	1.723	0.0864

**Table 5:** Multivariate regression analysis assessing the relationship between serum lactoferrin concentrations (ng/mL) and selected variables.

	β	SE	*t*	*p*
Total Population (*n* = 300)				
Model 1				
BMI (kg/m^2^)	0.188	0.062	3.019	0.0028
hs-CRP (mg/L)	0.036	0.062	0.572	0.5674
Model 2				
Waist circumference (cm)	0.179	0.060	2.990	0.0030
hs-CRP (mg/L)	0.057	0.060	0.945	0.3453
Model 3				
Fat mass (%)	0.115	0.061	1.873	0.0621
hs-CRP (mg/L)	0.129	0.061	2.113	0.0355
Model 4				
VAT mass (g)	0.127	0.061	2.094	0.0371
hs-CRP (mg/L)	0.104	0.061	1.717	0.0871

## Supplementary Materials

The following are available online at https://www.mdpi.com/2072-6643/11/9/1976/s1, Figure S1: Correlations between lactoferrin levels and body weight in the total population; Figure S2: Correlations between lactoferrin levels and BMI in the total population; Figure S3: Correlations between lactoferrin levels and waist circumference in the total population; Figure S4: Correlations between lactoferrin levels and fat mass in the total population; Figure S5: Correlations between lactoferrin levels and insulin concentrations in the total population; Figure S6: Correlations between lactoferrin levels and hs-CRP concentrations in the total population; Figure S7: Correlations between lactoferrin levels and body weight in MHO subjects; Figure S8: Correlations between lactoferrin levels and BMI in MHO subjects; Figure S9: Correlations between lactoferrin levels and waist circumference in MHO subjects; Figure S10: Correlations between lactoferrin levels and visceral adipose tissue (VAT) mass in MHO subjects.; Figure S11: Correlations between lactoferrin levels and glucose concentrations in MHO subjects; Figure S12: Correlations between lactoferrin levels and HDL-C concentrations in MHO subjects; Figure S13: Correlations between lactoferrin levels and waist circumference in MUHO subjects; Figure S14: Correlations between lactoferrin levels and hs-CRP concentrations in MUHO subjects.
